# Supplementary material for: Mutagenesis screen uncovers lifespan extension through integrated stress response inhibition without reduced mRNA translation
Source: Nat Commun. 2021 Mar 15;12:1678. doi: 10.1038/s41467-021-21743-x (PMC7960713; doi:10.1038/s41467-021-21743-x)
Supplement: Supplementary file 1 — Supplementary Information [file 41467_2021_21743_MOESM1_ESM.pdf]

Supplementary information for

Title:
Mutagenesis screen uncovers lifespan extension through integrated stress response inhibition without reduced mRNA translation

Authors:
Maxime J. Derisbourg[1,4], Laura E. Wester[1,4], Ruth Baddi[1], Martin S. Denzel[1,2,3]*

Affiliations:
[1]Max Planck Institute for Biology of Ageing
D-50931 Cologne, Germany

[2]CECAD - Cluster of Excellence
University of Cologne
D-50931 Cologne, Germany

[3]Center for Molecular Medicine Cologne (CMMC)
University of Cologne
D-50931 Cologne, Germany

[4]These authors contributed equally

*Corresponding author. Email: martin.denzel@age.mpg.de

This file contains:

Supplementary Figures 1 to 6
Supplementary Tables 1 to 4

a

Chromosome	Position	Nucleotide substitution	Gene name	AA substitution	AA position	Strain name
III	3016950	C/T	*daf-2*	L/F	162	MSD104
III	3016315	G/A	*daf-2*	V/M	181	MSD212
III	3000601	T/A	*daf-2*	M/K	1406	MSD305
III	3000581	C/T	*daf-2*	H/Y	1413	MSD167
III	3000560	G/A	*daf-2*	G/S	1420	MSD055
III	2997074	A/G	*daf-2*	Y/C	1805	MSD155

b

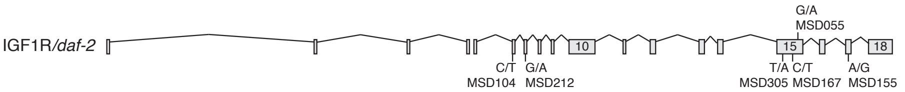

c

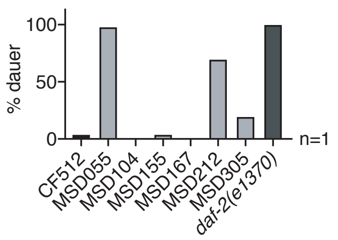

d

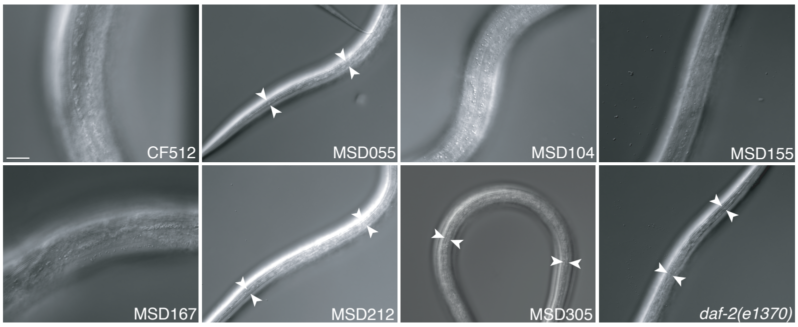

e

Chromosome	Position	Nucleotide substitution	Gene name	AA substitution	AA position	Strain name
I	8063756	T/A	*che-3*	N/I	3849	MSD121
I	8064225	G/A	*che-3*	S/F	3708	MSD202
I	8067069	C/T	*che-3*	A/T	3200	MSD097
I	8071899	T/A	*che-3*	E/V	2188	MSD215
IV	3798574	C/T	*osm-3*	A/T	140	MSD009
X	381554	C/T	*ife-2*	E/K	221	MSD218
X	382676	C/T	*ife-2*	G/E	10	MSD306
II	8220548	C/T	*ifg-1*	P/S	560	MSD295
II	8221040	G/A	*ifg-1*	E/K	685	MSD076

f

Chromosome	Position	Nucleitode substitution	Gene name	AA substitution	AA position	Allele name
II	6966468	T/A	*ppp-1*	N/I	295	*ppp-1(wrm10)II*
II	6966802	G/A	*ppp-1*	L/F	216	*ppp-1(wrm15)II*
II	13088136	G/A	*gcn-2*	D/N	1217	*gcn-2(wrm4)II*
X	11414450	C/T	*pek-1*	D/N	81	*pek-1(wrm7)X*
I	8026808	C/T	*iftb-1*	E/K	244	*iftb-1(wrm53)I*

Supplementary Fig. 1 | Mutations clustering in known longevity pathways and in the ISR identified through a mutagenesis screen for longevity. a Detailed overview of identified alleles in the *daf-2* insulin/insulin-like growth factor 1 (IGF-1) receptor gene. **b** Schematic representation of the *daf-2* gene and corresponding alleles identified in the screen, also shown in (**a**). **c** Percentage of worms entering the dauer stage upon development at 27 °C. Analyzed were CF512 controls, mutant strains from the screen carrying *daf-2* mutations (MSD strains shown in (**a**) and (**b**)), and a dauer constitutive (daf-c) *daf-2(e1370)* control (n=1 with ≥50 animals per genotype). **d** Microscopic analysis of dauer alae in the control and mutant worm strains depicted in (**a**), (**b**), and (**c**). White arrows indicate dauer alae. Scale bar is 20 μm, n=1. **e** Detailed overview of alleles in known longevity genes *che-3*, *osm-3*, *ife-2*, and *ifg-1* identified in the screen. **f** Detailed overview of identified longevity alleles clustering in the ISR identified in the screen. Source data are provided as a Supplementary Source Data file.

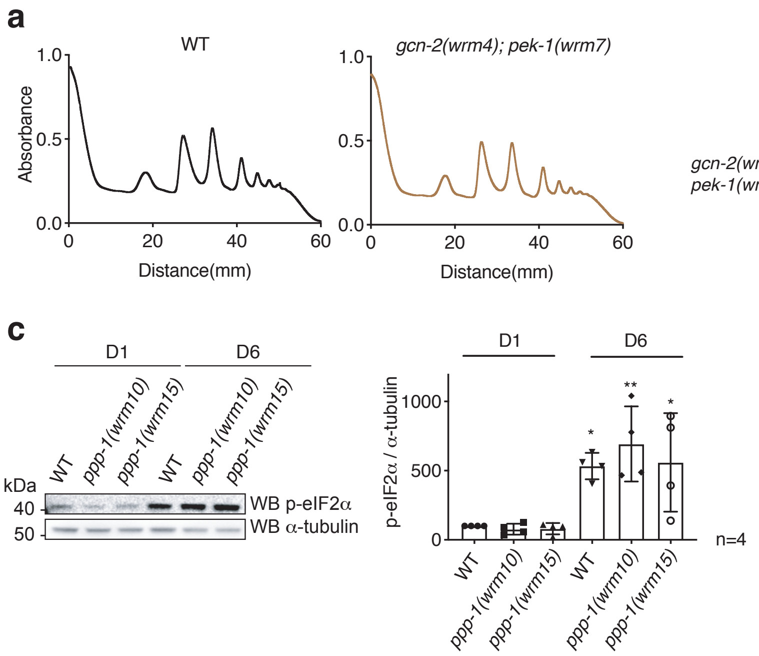

Supplementary Fig. 2 | Polysome profiling of ISR kinase mutants and ISR analysis in aged *ppp-1* mutants. a, b Polysome profiling and quantification of day 1 WT and *gcn-2(wrm4); pek-1(wrm7)* animals. Quantification represents the relative abundance of ribosomal subunits (40S, 60S), monosomes, and polysomes (error bars represent means +SD, two-way ANOVA Dunnett's post hoc test; n=3 independent experiments; no significant changes were detected). **c** Representative Western blot and quantification of day 1 and day 6 WT animals and *ppp-1* mutants detecting phospho-eIF2α (Ser51) normalized to α-tubulin (error bars represent means +SEM, one-way ANOVA Tukey's post hoc test with *p=0.0182 WT day 1 vs WT day 6; p=0,012 WT day 1 vs *ppp-1(wrm10)* day 6; **p<0.0014 WT day 1 vs *ppp-1(wrm15)* day 6; n=4 independent experiments). Source data are provided as a Supplementary Source Data file.

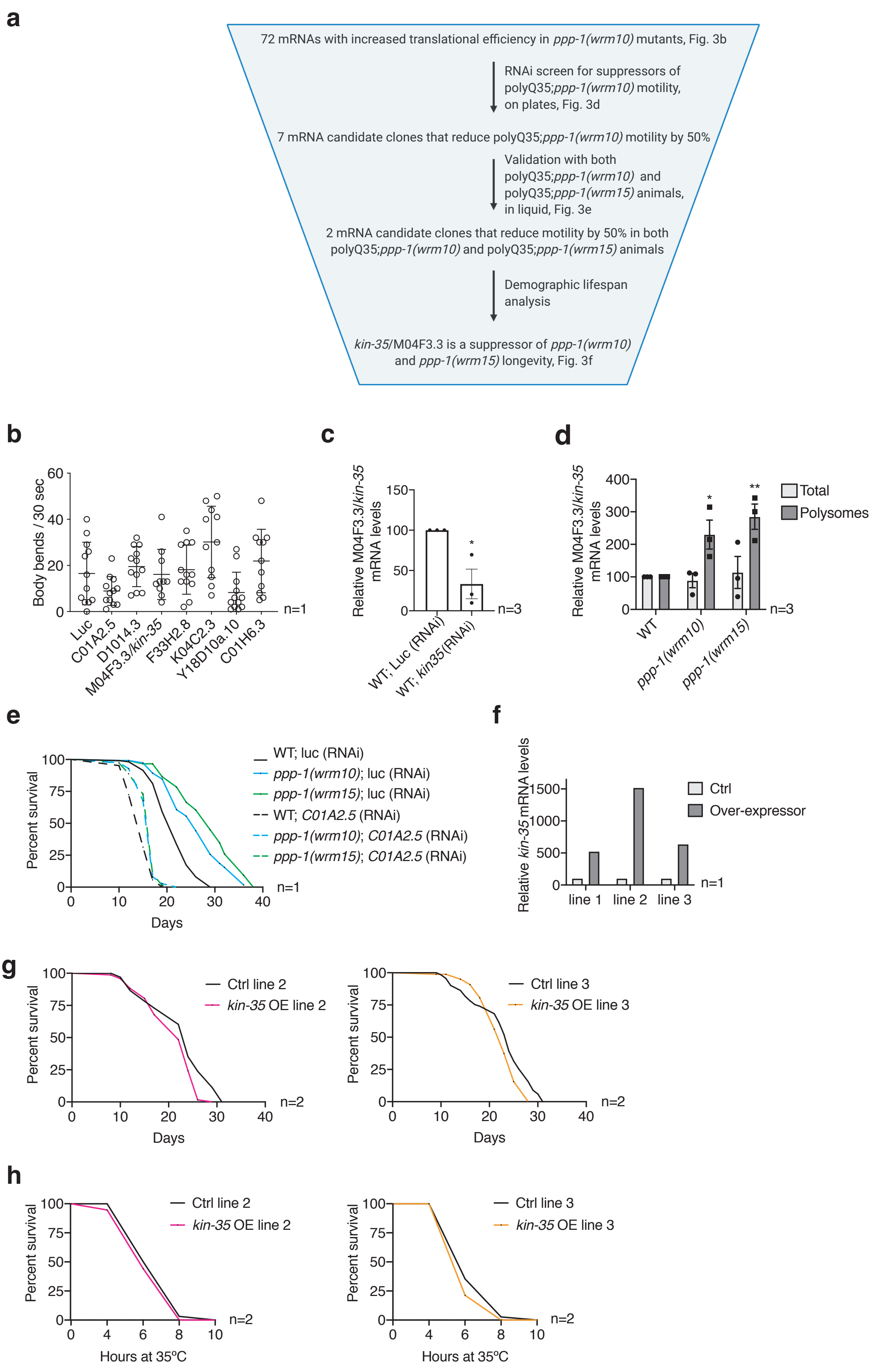

a
72 mRNAs with increased translational efficiency in ppp-1(wrm10) mutants, Fig. 3b
RNAi screen for suppressors of polyQ35;ppp-1(wrm10) motility, on plates, Fig. 3d
7 mRNA candidate clones that reduce polyQ35;ppp-1(wrm10) motility by 50%
Validation with both polyQ35;ppp-1(wrm10) and polyQ35;ppp-1(wrm15) animals, in liquid, Fig. 3e
2 mRNA candidate clones that reduce motility by 50% in both polyQ35;ppp-1(wrm10) and polyQ35;ppp-1(wrm15) animals
Demographic lifespan analysis
kin-35/M04F3.3 is a suppressor of ppp-1(wrm10) and ppp-1(wrm15) longevity, Fig. 3f
b
Body bends / 30 sec
Luc
C01A2.5
D1014.3
M04F3.3/kin-35
F33H2.8
K04C2.3
Y18D10a.10
C01H6.3
n=1
c
Relative M04F3.3/kin-35 mRNA levels
WT; Luc (RNAi)
WT; kin35(RNAi)
n=3
d
Relative M04F3.3/kin-35 mRNA levels
Total
Polysomes
WT
ppp-1(wrm10)
ppp-1(wrm15)
n=3
e
Percent survival
Days
WT; luc (RNAi)
ppp-1(wrm10); luc (RNAi)
ppp-1(wrm15); luc (RNAi)
WT; C01A2.5 (RNAi)
ppp-1(wrm10); C01A2.5 (RNAi)
ppp-1(wrm15); C01A2.5 (RNAi)
n=1
f
Relative kin-35 mRNA levels
Ctrl
Over-expressor
line 1
line 2
line 3
n=1
g
Percent survival
Days
Ctrl line 2
kin-35 OE line 2
Ctrl line 3
kin-35 OE line 3
n=2
h
Percent survival
Hours at 35°C
Ctrl line 2
kin-35 OE line 2
Ctrl line 3
kin-35 OE line 3
n=2

Supplementary Fig. 3 | Supporting data for the RNAi screen for suppressors of polyQ35; *ppp-1* motility. a Schematic representation of the RNAi screen for suppressors of polyQ35; *ppp-1* motility followed by verification and analysis of motility suppressors. **b** Control motility assays of day 6 polyQ35 transgenic worms after indicated RNAi treatments (error bars represent means ±SD, one-way ANOVA Dunnett's post hoc test, no significant changes were detected; n=1 with ≥10 worms per RNAi treatment). **c** qPCR for *kin-35* mRNA in WT animals using luciferase control or *kin-35* RNAi (error bars represent means ±SEM, unpaired two-tailed t-test with *p=0.0223 vs luciferase control; n=3 independent experiments). **d** mRNA distribution of *kin-35* mRNA in total worm extracts and polysomes of day 1 WT and *ppp-1* animals measured by qPCR (error bars represent means ±SEM, two-way ANOVA Tukey's post hoc test with *p=0.00381 and **p=0.0046 versus WT controls; n=3 independent experiments). **e** Survival of WT animals and *ppp-1* mutants upon RNAi knockdown of C01A2.5 and control *luciferase* (n=1). **f** Relative *kin-35* mRNA levels measured by qPCR in *kin-35* over-expressing worm lines 1, 2, and 3 compared to the respective non-transgenic littermate controls (n=1). **g** Survival of *kin-35* over-expressing worm lines 2 and 3 compared to respective control lines without the extrachromosomal array (representative data from n=2 independent experiments each). **h** Thermotolerance assays of day 1 *kin-35* over-expressing worm lines 2 and 3 compared to respective controls as described in (**g**) (representative data from n=2 independent experiments with ≥30 animals each). OE=Over-expressor. See Supplementary Dataset 1 for survival statistics. See Supplementary Table 1 for statistics on thermotolerance assays. Source data are provided as a Supplementary Source Data file.

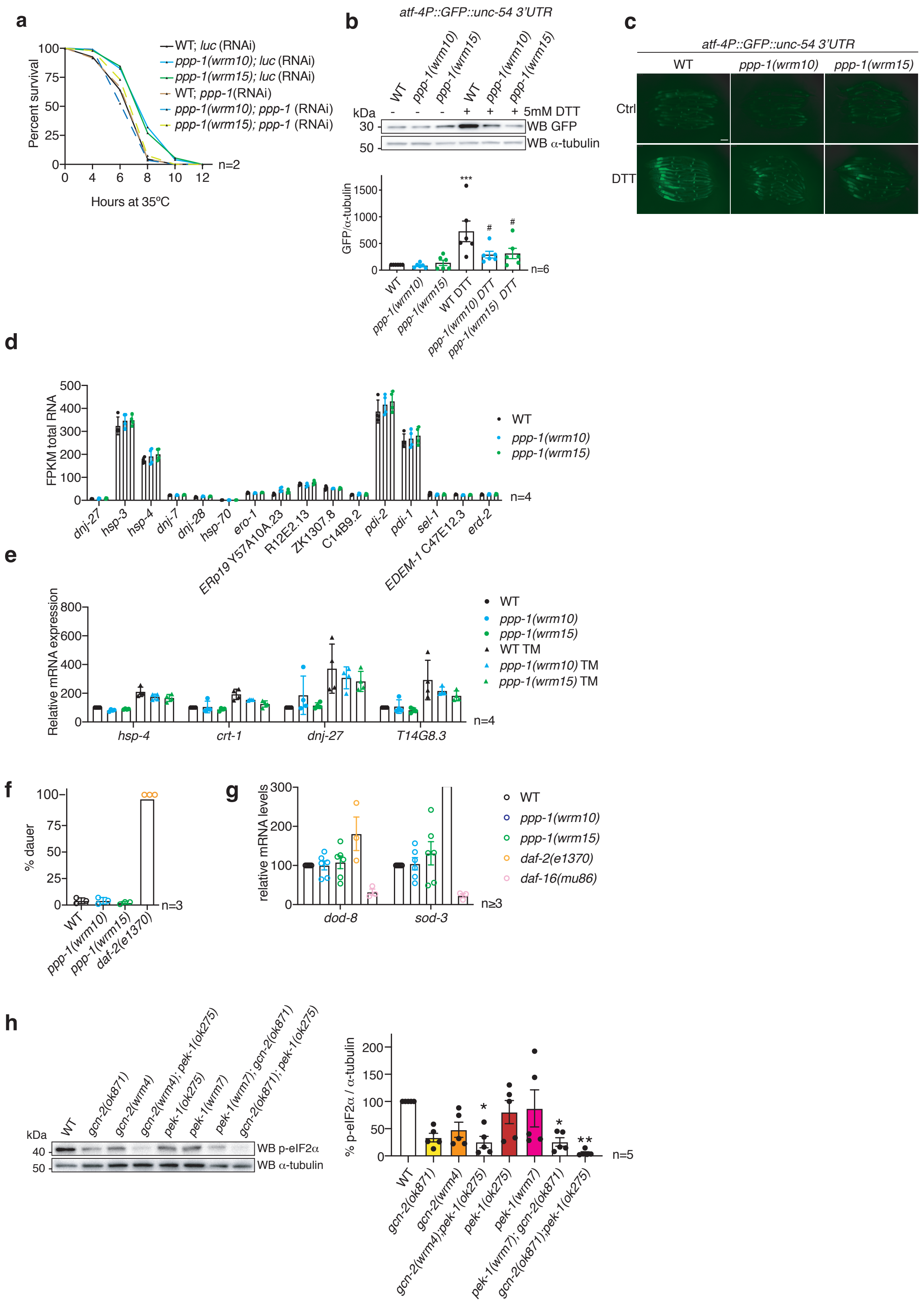

Supplementary Fig. 4 | Inhibiting the ISR through *ppp-1* mutations does not activate UPR or insulin signaling pathways. a Thermotolerance assays of day 1 WT animals and *ppp-1* mutants upon *ppp-1* RNAi treatment (representative data from n=2 independent

experiments with ≥30 worms each). **b** Representative Western blot of day 1 WT animals and *ppp-1* mutants in the *atf-4P*::GFP::*unc-54* 3'UTR reporter background treated with 5 mM DTT for 2 h, using anti-GFP and anti-α-tubulin antibodies. GFP levels were normalized to α-tubulin (error bars represent means +SEM, one-way ANOVA Tukey's post hoc test, ***p<0.0007 vs WT(-DTT), #p<0.0297 *ppp-1(wrm10)* vs WT(+DTT); #p<0.0438 *ppp-1(wrm15)* versus WT(+DTT); n=6 independent experiments). **c** Representative fluorescence images of day 1 WT animals and *ppp-1* mutants in the *atf-4P*::GFP::*unc-54* 3'UTR background, incubated without (Ctrl) or with 5 mM DTT for 2 h. Scale bar is 75 µm, n=3 independent experiments. **d** mRNA levels of indicated UPR genes measured by RNA sequencing in un-stressed WT and *ppp-1* animals depicted as fragments per kilo base per million mapped reads (FPKM; n=4 independent experiments). **e** Relative mRNA levels of indicated UPR genes measured by qPCR in WT animals and *ppp-1* mutants. Day 1 animals were treated with 1% DMSO control or 10 µg/mL tunicamycin (TM) for 6 h (n=4 independent experiments). **f** Percentage of worms entering the dauer stage upon development at 27 °C. Analyzed were WT, *ppp-1* and dauer constitutive *daf-2(e1370)* control animals (n=3 independent experiments with ≥30 animals each). **g** Relative mRNA levels of indicated *daf-16*/FOXO target genes measured by qPCR in WT animals, *ppp-1* mutants, and *daf-2(e1370)* and *daf-16(mu86)* controls (n≥3 independent experiments). **h** Representative Western blot and quantification of day 1 worms of indicated genotypes detecting phospho-eIF2α (Ser51) and α-tubulin. Levels of phospho-eIF2α were normalized to α-tubulin (error bars represent means +SEM, one-way ANOVA Dunnett's post hoc test, *p=0.0435 WT vs *gcn-2(wrm4); pek-1(ok275)*; *p=0.0445 WT vs *pek-1(wrm7); gcn-2(ok871);* **p=0.0445 WT vs *pek-1(ok275); gcn-2(ok871);* n=5 independent experiments). See Supplementary Table 1 for statistics on thermotolerance assays. Source data are provided as a Supplementary Source Data file.

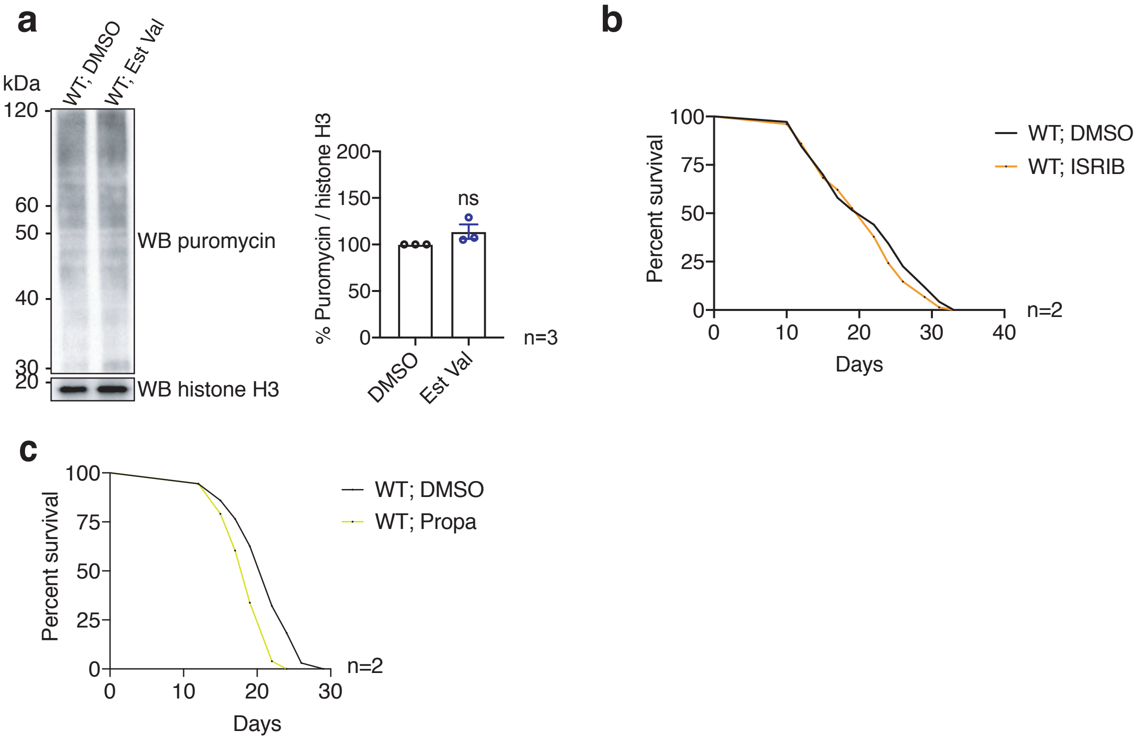

Supplementary Fig. 5 | Supplementary information on ISR-modulating compounds in *C. elegans*. a Puromycin incorporation followed by Western blot analysis using antibodies detecting puromycin and histone H3 in day 1 WT animals treated with DMSO (control) or 20 µM estradiol valerate (error bars represent means +SEM, unpaired two-tailed t-test; n=3 independent experiments; no significant changes were detected). **b** Survival of WT worms grown on NGM plates supplemented with 1% DMSO or 20 µM ISRIB (n=2 independent experiments). **c** Survival of WT worms grown on NGM plates supplemented with 1% DMSO or 20 µM propafenone hydrochloride (n=2 independent experiments). See Supplementary Dataset 1 for survival statistics. Source data are provided as a Supplementary Source Data file.

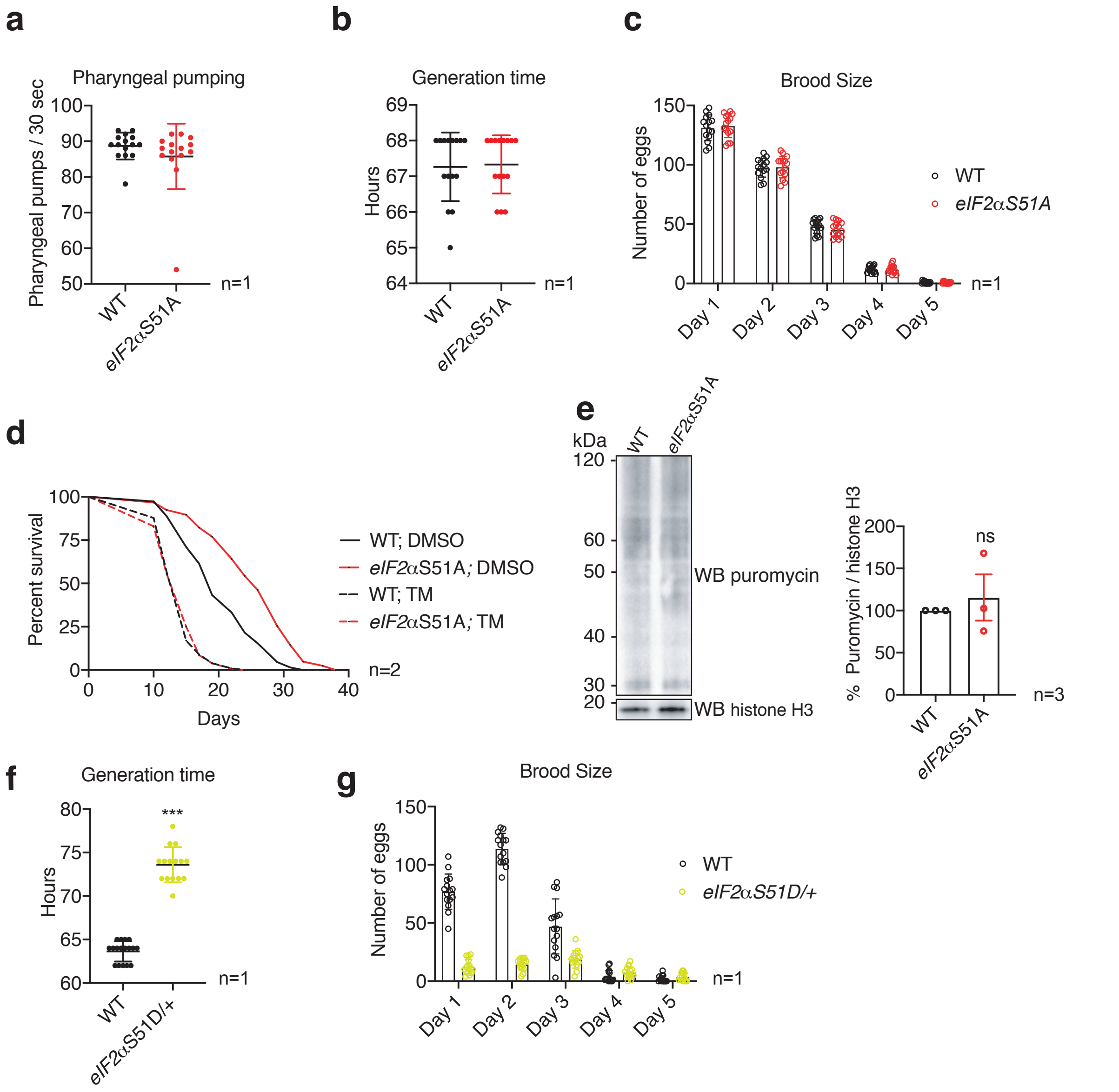

Supplementary Fig. 6 | Characterization of phospho-defective *eIF2α*S51A and heterozygous phospho-mimic *eIF2α*S51D mutants. a Pharyngeal pumping rates of day 1 WT animals and *eIF2α*S51A mutants (error bars represent means ±SD; n=1 with ≥15 animals per genotype). **b** Generation time of WT animals and *eIF2α*S51A mutants (error bars represent means ±SD; n=1 with ≥15 animals per genotype). **c** Brood size of WT and *eIF2α*S51A animals (error bars represent means +SD; n=1 with ≥15 animals per genotype). **d** Survival of WT animals and *eIF2α*S51A mutants treated with 1% DMSO (vehicle control) or 20 µg/mL tunicamycin (TM) from day 1 of adulthood (n=2 independent experiments). **e** Puromycin incorporation followed by Western blot analysis using antibodies detecting puromycin and histone H3 in day 1 WT animals and *eIF2α*S51A mutants (error bars represent means +SEM, unpaired two-tailed t-test; n=3 independent experiments; no significant changes were detected). **f** Generation time of WT animals and heterozygous phospho-mimic *eIF2α*S51D/+ mutants (error bars represent means ±SD, unpaired two-tailed t-test ***$p<0.0001$ versus WT;

n=1 with ≥15 animals per genotype). **g** Brood size of WT and heterozygous phospho-mimic *eIF2α*S51D/+ animals (error bars represent means +SD; n=1 with ≥15 animals per genotype). See Supplementary Dataset 1 for survival statistics. Source data are provided as a Supplementary Source Data file.

Supplementary Table 1 | Thermotolerance statistics.

(in percent live animals)

Fig. 1i

Time (Hours)	WT				*ppp-1(wrm10)* II				*ppp-1(wrm15) II*			
0	100	100	100	100	100	100	100	100	100	100	100	100
4	83	95	88	100	98	97	100	98	96	100	100	100
6	50	53	40	32	86	77	94	70	82	68	87	72
8	0	0	0	0	22	14	30	24	36	15	49	24
10	0	0	0	0	0	0	0	8	0	0	0	4
12	0	0	0	0	0	0	0	0	0	0	0	0

Fig. 3h and Supplementary 3h

Time (Hours)	Ctrl line 1		*kin-35* OE line 1		Ctrl line 2		*kin-35* OE line 2		Ctrl line 3		*kin-35* OE line 3	
0	100	100	100	100	100	100	100	100	100	100	100	100
4	100	100	100	100	100	100	92	97	100	100	100	100
6	55	30	35	27	44	56	57	32	35	36	23	19
8	3	0	5	0	0	6	0	0	5	0	0	0
10	0	0	0	0	0	0	0	0	0	0	0	0

Supplementary Fig. 4a

	Luciferase (RNAi)						ppp-1 (RNAi)					
Time (Hours)	WT		*ppp-1(wrm10)* II		*ppp-1(wrm15) II*		WT		*ppp-1(wrm10)* II		*ppp-1(wrm15) II*	
0	100	100	100	100	100	100	100	100	100	100	100	100
4	91	94	100	98	100	96	89	94	98	100	96	100
6	67	61	75	90	87	82	67	66	45	60	70	76
8	9	0	33	31	26	28	9	0	7	0	9	6
10	0	0	4	4	7	4	0	0	0	0	0	0
12	0	0	0	0	0	0	0	0	0	0	0	0

Fig. 6d

Time (Hours)	WT				*eIF2a (syb1385) I*			
0	100	100	100	100	100	100	100	100
4	77	88	88	81	81	83	94	98
6	15	15	38	27	60	63	63	58
8	0	0	0	0	15	15	17	17
10	0	0	0	0	4	6	2	4
12	0	0	0	0	0	0	0	0

Supplementary Table 2 | Worm strains used in this study.

Strain name	Genotype	Backcrossed to N2	Source
MSD331	Bristol N2		CGC
CF512	*fer15(b26) II; fem-1(hc17) IV*	n/a	CGC
MSD283	*ppp-1(wrm10) II*	4x	this study
MSD310	*ppp-1(wrm15) II*	4x	this study
SYB691	*ppp-1(syb691) II* CR-N295I (wrm10)	n/a	Suny Biotech
SYB728	*ppp-1(syb728) II* CR-L216F (wrm15)	n/a	Suny Biotech
MSD513	*iftb-1(wrm53) I*	4x	this study
MSD275	*gcn-2(wrm4) II*	4x	this study
MSD302	*pek-1(wrm7) X*	4x	this study
MSD413	*gcn-2(wrm4) II; pek-1(wrm7) X*	n/a	this study
MSD535	*gcn-2(ok871) II*	2x	this study
MSD511	*pek-1(ok275) X*	2x	this study
MSD412	*gcn-2(ok871) II; pek-1(ok275) X*	2x	this study
MSD512	*gcn-2(wrm4) II; pek-1(ok275) X*	n/a	this study
MSD536	*gcn-2(ok871) II; pek-1(wrm7) X*	n/a	this study
MSD441	*mIs133[unc-54p::Q35:YFP]*	2x	CGC
MSD442	*mIs133[unc-54p::Q35:YFP]; ppp-1(wrm10)*	2x	this study
MSD457	*mIs133[unc-54p::Q35:YFP]; ppp-1(wrm15)*	2x	this study
MSD505	*pkIs2386[Punc-54::alpha-synuclein::YFP; unc-119(+)]*	2x	this study
MSD506	*pkIs2386[Punc-54::alpha-synuclein::YFP; unc-119(+)]; ppp-1(wrm10)*	2x	this study
MSD507	*pkIs2386[Punc-54::alpha-synuclein::YFP; unc-119(+)]; ppp-1(wrm15)*	2x	this study
MSD406	*rsks-1(sv31) III*	2x	Hubbard lab
MSD538	*sybEx[M04F3.3P::M04F3.3::mCherry;unc-54 3'UTR]* line 1	n/a	Suny Biotech
MSD539	*sybEx[M04F3.3P::M04F3.3::mCherry;unc-54 3'UTR]* line 2	n/a	Suny Biotech
MSD540	*sybEx[M04F3.3P::M04F3.3::mCherry;unc-54 3'UTR]* line 3	n/a	Suny Biotech
MSD314	*ldIs(T04C10.4/atf-4P::GFP::unc-54 3'UTR)*	2x	Blackwell lab
MSD317	*ldIs(T04C10.4/atf-4P::GFP::unc-54 3'UTR); ppp-1(wrm10) II*	2x	this study
MSD456	*ldIs(T04C10.4/atf-4P::GFP::unc-54 3'UTR); ppp-1(wrm15) II*	2x	this study
SYB1385	*Y37E3.10a(syb1385) I*	2x	Suny Biotech
PHX1567	*Y37E3.10a(syb1567)/hT2[bli-4(e937)let-?(q782)qIs48] I*	2x	Suny Biotech
MSD498	*daf-2(e1370) III*	4x	this study
MSD340	*daf-16(mu86) I*	4x	this study
MSD104	EMS mutant strain containing a *daf-2* mutation	n/a	this study
MSD212	EMS mutant strain containing a *daf-2* mutation	n/a	this study
MSD305	EMS mutant strain containing a *daf-2* mutation	n/a	this study
MSD167	EMS mutant strain containing a *daf-2* mutation	n/a	this study
MSD055	EMS mutant strain containing a *daf-2* mutation	n/a	this study
MSD155	EMS mutant strain containing a *daf-2* mutation	n/a	this study
MSD121	EMS mutant strain containing a *che-3* mutation	n/a	this study
MSD202	EMS mutant strain containing a *che-3* mutation	n/a	this study
MSD097	EMS mutant strain containing a *che-3* mutation	n/a	this study
MSD215	EMS mutant strain containing a *che-3* mutation	n/a	this study
MSD009	EMS mutant strain containing an *osm-3* mutation	n/a	this study
MSD218	EMS mutant strain containing an *ife-2* mutation	n/a	this study
MSD306	EMS mutant strain containing an *ife-2* mutation	n/a	this study
MSD295	EMS mutant strain containing an *ifg-1* mutation	n/a	this study
MSD076	EMS mutant strain containing an *ifg-1* mutation	n/a	this study

Supplementary Table 3 | Genotyping methods and PCR primers used in this study.

Genotype	Genotyping Method	Primer sequence
ppp-1(wrm10) II<br>*ppp-1(wrm15) II*<br>*gcn-2(wrm4) II*<br>*pek-1(wrm7) X*	TaqMan SNP mapping	Manufactured by Applied Biosysems
ppp-1(syb691) II CR-N295I (wrm10)	PCR	For TCCTTGTCAATCTGAATGGA<br>Rev CCATCGATCAAATTAACTTCA
ppp-1(syb728) II CR-L216F (wrm15)	PCR	For GCAGTTACAAATCCATTTT<br>Rev CCTTAATAATTCTAATTTTTACA
iftb-1(wrm53) I	PCR	CGTAATGTGCCATACTTGCAAG<br>CTCAAAACACGAATCGAATGAG
gcn-2(ok871) II	PCR	For GCATGACATGGCAATGATTCATCG<br>Rev GTACTTTGCAATGATTTTGAGGCG
pek-1(ok275) X	PCR	For CTGAGAAGGCAACGCTCTCT<br>Rev ATCACCGCTACTCTGGATGG
eIF2α(syb1385) I	PCR	For ATTGGTAGTTTATCTCATTTAAT<br>Rev TTCTCCTTAATATCTGCACT

Supplementary Table 4 | RT-qPCR primers used in this study.

Target gene	RT-qPCR primer sequence
act-1	For CTACGAACTTCCTGACGGACAAG<br>Rev CCGGCGGACTCCATACC
kin-35	For GGTTGAATATTGGTGAGGAGGTTGT<br>Rev TGCCACCATGATCTCTCTTTCAATC
hsp-4	For GTGGCAAACGCGTACTGTGATGA<br>Rev CGCAACGTATGATGGAGTGATTCT
crt-1	For TGTGGCAGGTCAAGTCAGGAAC<br>Rev TTTCTTCGTCGGCCTTCTCCTTC
dnj-27	For GAGAGCTTGCAGAGGTTATTGGAG<br>Rev GCATCATTGGCAAGCATCCATACG
T14G8.3	For GGATCAAGCTAATGAGCAGCAGAC<br>Rev TGACAGGTTTACAGTTCCGATGC
dod-8	For ACAGGATGTCTTCAAAAGGAATATGG<br>Rev TTGCTGGGGTGATAGCTTGG
sod-3	For CACGAGGCTGTTTCGAAAGG<br>Rev GAATTTCAGCGCTGGTTGGA
