# Supplementary material for: Mutagenesis screen uncovers lifespan extension through integrated stress response inhibition without reduced mRNA translation
Source: Nat Commun. 2021 Mar 15;12:1678. doi: 10.1038/s41467-021-21743-x (PMC7960713; doi:10.1038/s41467-021-21743-x)
Supplement: Supplementary file 3 — Description of Additional Supplementary Files [file 41467_2021_21743_MOESM3_ESM.pdf]

Description of Additional Supplementary Files

File Name: Supplementary Data 1

Description: Survival statistics (Mantel-Cox log rank method).

File Name: Supplementary Data 2

Description: List of polysome-associated mRNAs in WT animals and ppp-1 mutants. Data displayed as fold change in WT versus ppp-1. To analyze the translatome, the abundance of each mRNA in the polysomal fraction was normalized to its abundance in the total input mRNA. Respective normalized values were used to identify changes between different conditions using two-sided Student's t-test. For further analyses, we only included the mRNAs that were found significantly changed in both ppp-1 mutant alleles. For each mRNA, the mean p-values and the mean log-2 fold change of both ppp-1 mutants were used
