# Supplementary material for: Mutagenesis screen uncovers lifespan extension through integrated stress response inhibition without reduced mRNA translation
Source: Nat Commun. 2021 Mar 15;12:1678. doi: 10.1038/s41467-021-21743-x (PMC7960713; doi:10.1038/s41467-021-21743-x)
Supplement: Supplementary file 6 — Reporting Summary [file 41467_2021_21743_MOESM6_ESM.pdf]

nature research

Corresponding author(s): Martin S. Denzel

Last updated by author(s): Dec 21, 2020

Reporting Summary

Nature Research wishes to improve the reproducibility of the work that we publish. This form provides structure for consistency and transparency in reporting. For further information on Nature Research policies, see Authors & Referees and the Editorial Policy Checklist .

Statistics

For all statistical analyses, confirm that the following items are present in the figure legend, table legend, main text, or Methods section.

n/a	Confirmed	
☐	☒	The exact sample size (*n*) for each experimental group/condition, given as a discrete number and unit of measurement
☐	☒	A statement on whether measurements were taken from distinct samples or whether the same sample was measured repeatedly
☐	☒	The statistical test(s) used AND whether they are one- or two-sided<br>*Only common tests should be described solely by name; describe more complex techniques in the Methods section.*
☒	☐	A description of all covariates tested
☐	☒	A description of any assumptions or corrections, such as tests of normality and adjustment for multiple comparisons
☐	☒	A full description of the statistical parameters including central tendency (e.g. means) or other basic estimates (e.g. regression coefficient) AND variation (e.g. standard deviation) or associated estimates of uncertainty (e.g. confidence intervals)
☐	☒	For null hypothesis testing, the test statistic (e.g. *F*, *t*, *r*) with confidence intervals, effect sizes, degrees of freedom and *P* value noted<br>*Give P values as exact values whenever suitable.*
☒	☐	For Bayesian analysis, information on the choice of priors and Markov chain Monte Carlo settings
☒	☐	For hierarchical and complex designs, identification of the appropriate level for tests and full reporting of outcomes
☒	☐	Estimates of effect sizes (e.g. Cohen's *d*, Pearson's *r*), indicating how they were calculated

Our web collection on statistics for biologists contains articles on many of the points above.

Software and code

Policy information about availability of computer code

Data collection	Leica Application Suite X (Version 3.4.1.17822)<br>ImageLab Software (version 5.2, Biorad)
Data analysis	CloudMap Unmapped Mutant Workflow pipeline on Galaxy<br>Prism (version 8.2.0)<br>Zen 2.3 pro software<br>ImageJ (version 1.51)<br>HISAT2 (v2.1.0)<br>StringTie (v1.3.4d)<br>Cufflinks (v2.2.1)

For manuscripts utilizing custom algorithms or software that are central to the research but not yet described in published literature, software must be made available to editors/reviewers. We strongly encourage code deposition in a community repository (e.g. GitHub). See the Nature Research guidelines for submitting code & software for further information.

Data

Policy information about availability of data

All manuscripts must include a data availability statement. This statement should provide the following information, where applicable:

- Accession codes, unique identifiers, or web links for publicly available datasets
- A list of figures that have associated raw data
- A description of any restrictions on data availability

The RNA sequencing data in this publication have been deposited in NCBI’s Gene Expression Omnibus and are accessible through GEO Series accession number GSE144607 (https://www.ncbi.nlm.nih.gov/geo/query/acc.cgi?acc=GSE144607). All other data is available in the main text or the supplementary materials. The

source data underlying Figs. 1j, 1k, 2a-e, 4d, 5c, 6a, 6b, 6e, 6f and Supplementary Figs. 1c, 2a-c, 3b-e, 4b, 4e-h, 5a, 6a-c and 6e-g are provided as a Source Data file.

Field-specific reporting

Please select the one below that is the best fit for your research. If you are not sure, read the appropriate sections before making your selection.

[x] Life sciences [ ] Behavioural & social sciences [ ] Ecological, evolutionary & environmental sciences

For a reference copy of the document with all sections, see nature.com/documents/nr-reporting-summary-flat.pdf

Life sciences study design

All studies must disclose on these points even when the disclosure is negative.

Sample size	All C. elegans sample sizes were chosen based on standard C. elegans technique protocols publicly available, tested and, if necessary, modified to gain results of sufficient quality. Sample size for survival assays was done with at least 100 animals.
Data exclusions	In all lifespan and survival experiments, worms that had undergone internal hatching, vulval bursting, or worms crawling off the plates were censored. These are pre-established exclusion criteria well accepted in the field.
Replication	All experiments have been replicated and the number of independent repeats is in the figure legends and in the supplementary tables.
Randomization	C. elegans animals are selected randomly for experimentation
Blinding	All experiments measuring C. elegans behavioural traits and survival were blinded. Experiments with indirect output like Western blots, polysome profiling, RNA sequencing, 35S-methionine labelling, and qPCRs were not blinded.

Reporting for specific materials, systems and methods

We require information from authors about some types of materials, experimental systems and methods used in many studies. Here, indicate whether each material, system or method listed is relevant to your study. If you are not sure if a list item applies to your research, read the appropriate section before selecting a response.

Materials & experimental systems

n/a	Involved in the study	
[ ]	[x]	Antibodies
[x]	[ ]	Eukaryotic cell lines
[x]	[ ]	Palaeontology
[ ]	[x]	Animals and other organisms
[x]	[ ]	Human research participants
[x]	[ ]	Clinical data

Methods

n/a	Involved in the study	
[x]	[ ]	ChIP-seq
[x]	[ ]	Flow cytometry
[x]	[ ]	MRI-based neuroimaging

Antibodies

Antibodies used	Phospho-eIF2α (Ser51) (clone D9G8, Cell Signaling #3398), Anti-Puromycin (clone 12D10, Merck Millipore #MABE343), Living Colors GFP (Clontech), α-Tubulin (clone DM1A, Sigma #T9026), Histone H3 (Abcam #AB1791)
Validation	Phospho-eIF2α (Ser51): Validated in stress assays that enhance phosphorylation, in ISR kinase mutant strains, and in a eIF2αS51A mutant that lacks the specific phosphorylation-site<br>Anti-Puromycin: Validated in control samples with low translation or without puromycin treatment<br>Living Colors GFP: Validated in non-transgenic negative control samples<br>α-Tubulin and Histone H3: Used as loading control markers

Animals and other organisms

Policy information about studies involving animals; ARRIVE guidelines recommended for reporting animal research

Laboratory animals	Caenorhabditis elegans, which are hermaphrodites Experiments such as Western blots, RNA and polysome sequencing were done at the age of day 1 of adulthood unless stated otherwise. Lifespan and thermotolerance assays were performed during the whole life of the animals.
Wild animals	No wild animals were used in the study.
Field-collected samples	No field collected samples were used in the study.

Ethics oversight	The study did not require ethical approval.

Note that full information on the approval of the study protocol must also be provided in the manuscript.
